# Effects of 8-Week Hatha Yoga Training on Metabolic and Inflammatory Markers in Healthy, Female Chinese Subjects: A Randomized Clinical Trial

**DOI:** 10.1155/2016/5387258

**Published:** 2016-08-03

**Authors:** Neng Chen, Xianghou Xia, Liqiang Qin, Li Luo, Shufen Han, Guiping Wang, Ru Zhang, Zhongxiao Wan

**Affiliations:** ^1^Department of Nutrition and Food Hygiene, School of Public Health, Soochow University, 199 Renai Road, Suzhou 215123, China; ^2^Department of Breast Surgery, Zhejiang Cancer Hospital, Hangzhou, Zhejiang 310022, China; ^3^School of Physical Education and Sports Science, Soochow University, Suzhou 215006, China; ^4^Laboratory Animal Center, Soochow University, 199 Renai Road, Suzhou 215123, China; ^5^Jiangsu Key Laboratory of Preventive and Translational Medicine for Geriatric Disease, Soochow University, 199 Renai Road, Suzhou 215123, China

## Abstract

We aimed to determine the effects of an 8 wk Hatha yoga training on blood glucose, insulin, lipid profiles, endothelial microparticles (EMPs), and inflammatory status in healthy, lean, and female Chinese subjects. A total of 30 healthy, female Chinese subjects were recruited and randomized into control or yoga practice group. The yoga practice included 8 wks of yoga practice (2 times/wk) for a total of 16 times. Fasting blood samples were collected before and after yoga training. Plasma was isolated for the measurement of lipid profiles, glucose, insulin, EMPs, and inflammatory cytokines. Whole blood was cultured* ex vivo* and stimulated with lipopolysaccharide (LPS) and Pam3Cys-SK4. Peripheral blood mononuclear cells (PBMCs) were isolated for the measurement of TLR2 and TLR4 protein expression. Yoga practice significantly reduced plasma cholesterol, LDL-cholesterol, insulin levels, and CD31+/CD42b− EMPs. Cultured whole blood from the yoga group has reduced proinflammatory cytokines secretion both at unstimulated condition and when stimulated with Pam3Cys-SK4; this might be associated with reduced TLR2 protein expression in PBMCs after yoga training. Hatha yoga practice in healthy Chinese female subjects could improve hallmarks related to MetS; thus it can be considered as an ancillary intervention in the primary MetS prevention for the healthy population. This trial is registered with ChiCTR-IOR-14005747.

## 1. Introduction

Yoga is a mind-body therapy that has become increasingly popular worldwide. Accumulating evidence suggests that yoga meditation could improve risk factors associated with metabolic syndrome (MetS) such as obesity, disordered lipid profile [1], and insulin resistance [2–4]. However, most of these studies are conducted in clinical populations [1–4] and there are surprisingly few studies examining how yoga training affects MetS' related risk factors in healthy subjects [5, 6]. In regard to this, Manjunatha et al. [5] reported that 5 days of yoga asanas increased the sensitivity of the *β* cells to the glucose signal in healthy subjects. Bhattacharya et al. [6] found that yoga practice can improve the antioxidant status of the healthy individual.

Endothelial microparticles (EMPs) are complex vesicular structures shed from endothelial cells in response to stimuli such as inflammatory activation [7]. They are now considered as novel biomarkers of endothelial activation and damage that are increased in overweight/obese individuals at risk for MetS [8, 9]. Evidence suggests that EMPs change with alterations in physical activity (PA) [10–12]. For example, reduced daily PA by taking <5,000 steps/day with a total of 5 days resulted in elevated CD31+/CD42b− EMPs in recreationally active men [12]. Similarly, enforced physical inactivity by subjecting healthy men to 7 days of dry immersion also led to increased circulating CD31+/CD41− EMPs [10]. In contrast, 6 months of supervised aerobic exercise training with moderate intensity could improve circulating EMPs levels as evidenced by decreased CD31+/CD42− EMPs in sedentary African American adults [11]. As yoga practice altered the blood flow velocity and consequently shear stress at the vascular wall [13], presumably it could affect EMPs. However, currently, there is no evidence whether yoga practice could affect EMPs, especially in Chinese subjects.

Inflammation is one of the key mechanisms involved in the pathogenesis of MetS [14]. Presently evidence examining effects of yoga on inflammatory processes is limited. Existing evidence suggests that yoga could positively affect circulating inflammatory markers in heart failure patients [15, 16], breast cancer survivors [17, 18], and patients with chronic inflammatory diseases and overweight/obese subjects [19]. Meanwhile, mind-body interventions that include some elements of yoga can reduce inflammatory signaling through NF-*κ*B pathway [17, 20, 21]. Toll-like receptors (TLRs), especially TLR2 and TLR4, play critical roles in innate immunity and may be involved in the link between physical activity, inflammation, and MetS [22–24]. However, it remains unclear whether yoga practice could affect circulating TLR2 and/or TLR4 response in healthy subjects.

Hatha yoga is the most commonly practiced worldwide. The key components of Hatha yoga are stretching exercises and physical postures, breath control, and concentration and thinking techniques designed to promote the well-being of the subjects both physically and emotionally [25]. With the above points in mind, the primary aim of the present study is to determine the effects of an 8 wk Hatha yoga practice on metabolic markers including blood glucose, insulin, lipid profiles, blood pressure, and EMPs in healthy, lean, and female Chinese subjects; the secondary aim is to determine the effects of Hatha yoga practice on inflammatory markers in the above subjects via measuring circulating cytokine levels, LPS, and Pam3Cys-SK4 (Pam) stimulated cytokines secretion in whole blood culture* ex vivo*, as well as TLR2 and TLR4 protein expression in PBMCs.

## 2. Materials and Methods

### 2.1. Materials

RPMI-1640, LPS (cat. number L6529-1) and 900 nm Latex beads carboxylate modified polyester (CLB9) were from Sigma (MO, USA). A custom human Adipokine Panel 2 (cat. number HADK2MAG-61K) containing primary and detection antibodies for interleukin- (IL-) 6, IL-8, IL-1*β*, monocyte chemoattractant protein- (MCP-) 1, tumor necrosis factor- (TNF-) *α*, and insulin was purchased from Merck Millipore (MA, USA). Pam (cat. number tlrl-pms) was from InvivoGen (CA, USA). Human IL-6 (cat. number DY206), IL-1*β* (cat. number DY201), and TNF-*α* (cat. number DY210) ELISA kit (DuoSet) was from R&D Systems (NE, USA). Antibodies against CD42b-PE (cat. number 555473), CD31-FITC (cat. number 555445), and CD62E-APC (cat. number 551144) were from BD Biosciences (NJ, USA). An antibody against TLR2 (cat. number 12276) was from Cell Signaling Technologies (MA, USA). TLR4 antibody (cat. number MAB1478) was from R&D Systems (NE, USA). All other chemicals were purchased from Sigma (MO, USA) unless otherwise noted.

### 2.2. Trial Design and Changes after Trial Commencement

This investigation reports a single-arm parallel-randomized controlled trial comparing the effects of 8 weeks of yoga intervention on metabolic and inflammatory markers in healthy female subjects. Ethical approval was obtained from the Human Research and Ethical Committee of the Soochow University and all participants provided signed informed consent. All methods were performed following the approved guidelines and regulations. This trial was registered in the Chinese Clinical Trial Registry with the number ChiCTR-IOR-14005747 on December 27, 2014. No changes to the methodology occurred following trial commencement. The data were reported according to the CONSORT statement [26].

### 2.3. Participants, Eligibility Criteria, and Settings

This study was conducted at School of Public Health, Soochow University, Jiangsu Province, China. Participants were recruited from the Campus of Soochow University via poster advertisement. The study inclusion criteria included age 18–25 years old; BMI > 18.5 and <23.9 kg/m^2^; the blood glucose, triacylglycerol, cholesterol, HDL-C, LDL-C, systolic blood pressure (SBP), and diastolic blood pressure (DBP) being within the normal ranges; and self-reported regular menstrual cycles (i.e., cycle 24–36 days long and at least 10 cycles in the previous 12 months). The exclusion criteria included subjects having history for using of pharmacologic contraceptives (past 6 months) and history of breast cancer, heart diseases, diabetes mellitus, or other serious medical conditions and subjects suffering from musculoskeletal conditions that would prevent participation in a yoga training.

### 2.4. Interventions

A total of 30 female subjects were recruited and randomized into control or yoga practice group. Participants in the yoga group were then asked to attend supervised Hatha yoga sessions 2 times per week over the 8 wks of the study. Yoga classes were offered on Monday and Thursday every week (from 6 p.m. to 7 p.m.). Each class has a total of 60 minutes and had the following components: breathing exercise (6 mins); loosening exercise (i.e., corn tree pose) (10 mins); standing poses (i.e., warrior pose and mountain pose) (8 mins); supine poses (i.e., bridge pose and dolphin plank pose) (8 mins); prone poses (i.e., hare pose and locust pose) (8 mins); sitting poses (i.e., staff pose and hero pose) (8 mins); relaxation/corpse pose (6 mins); and seated meditation (6 mins). Approximately 32 minutes is spent in active poses. The classes were held in a yoga training room and taught by a registered, specialized yoga instructor. The yoga practice was specifically designed for this study; however the yoga classes were not observed by study staff. Subjects were also instructed to maintain their usual physical activity and dietary habits for the study.

### 2.5. Primary and Secondary Outcomes

On day 1 of the study and 2 days after the whole yoga practice, subjects reported to the laboratory after an overnight fast; a baseline and a final blood sample (10 mL), respectively, were obtained by venipuncture from an antecubital vein and collected into EDTA tubes. Blood (9 mL) was centrifuged at 1500 g for 10 mins at 4°C and plasma was immediately frozen at −80°C for subsequent batch analyses of plasma cytokines, clinical biomarkers (i.e., insulin, glucose, triacylglycerol (TG), HDL-cholesterol, LDL-cholesterol, and total cholesterol), and endothelial microparticles. About 1 mL blood was utilized for whole blood culture. The height, body weight, SBP, and DBP of the subjects were measured by trained research assistants following standardized procedures using calibrated equipment.

The primary outcome measure for this trial was plasma insulin level, while secondary outcomes were (1) other clinical biomarkers (i.e., glucose, TG, HDL-cholesterol, LDL-cholesterol, and total cholesterol); (2) EMPs; and (3) plasma cytokines and cytokines from culture whole blood* ex vivo*. There were no changes to outcomes following trial commencement.

### 2.6. Sample Size Calculation

The sample size was based on (1) published findings from other research groups who have reported the beneficial effects of yoga with similar sample size [5, 6, 15] and (2) calculations assuming two-tailed *α* = 0.05 and 1-*β* = 90% to detect a 10% difference for the plasma insulin levels, which is the primary outcome of the present study.

### 2.7. Randomization and Blinding

Following recruitment randomization was carried out via computer-generated random numbers with unrestricted equal participant allocation (1 : 1) by one research investigator, who is independent of the yoga intervention and data analysis. Participants were not blinded to the study.

### 2.8. Plasma Clinical Metabolic Biomarkers Measurement

Clinical biomarkers including glucose, TG, HDL-cholesterol, LDL-cholesterol, and total cholesterol were measured on an automatic analyzer (Hitachi 7600, Tokyo, Japan). The homeostasis model assessment of insulin resistance (HOMA-IR) was calculated using the following equation: HOMA-IR = fasting insulin (FIns, *μ*IU/mL) × fasting blood glucose (FBG, mmol/L)/22.5.

### 2.9. Plasma Cytokines and Insulin Measurement

Plasma cytokines including IL-6, IL-8, IL-1*β*, MCP-1, TNF-*α*, and plasma insulin were measured from EDTA plasma using Luminex® technology according to the kit manufacturer's instructions. The detection limits for IL-6, IL-8, IL-1*β*, MCP-1, TNF-*α*, and insulin were 0.2, 0.3, 0.4, 1.2, 0.3, and 3.8 pg/mL, respectively. Plasma IL-6 and IL-1*β* levels were below the detection limit of the assay in our study. The average CV for duplicates in the assay is <6%.

### 2.10. Endothelial Microparticles (EMPs) Measurement

Circulating EMPs were measured in platelet-poor plasma by flow cytometry following the method of Jenkins et al. [27]. In brief, frozen plasma samples were thawed at room temperature for 20 minutes and centrifuged at 1500 g for 15 minutes. The top two-thirds of plasma were then further centrifuged at 1500 g for another 15 minutes to obtain platelet-poor plasma. The top 100 *μ*L of platelet-poor plasma was then incubated with fluorochrome labeled antibodies specific for PE-CD42b, FITC-CD31, and APC-CD62E for 20 minutes in the dark at 4°C. Samples were then fixed with 93 *μ*L of 2% paraformaldehyde and diluted up to 500 *μ*L with sterile, 0.2 *μ*M filtered PBS and analyzed on a FC500 Beckman Coulter (CA, USA). A microparticle size gate was determined using 900 nm Latex beads carboxylate modified polyester. Unstained and fluorescence minus one controls were used to differentiate between true events and background/debris. EMPs were identified as CD62E+ and CD31+/CD42b− events within the microparticle size gate.

### 2.11. Whole Blood Culture

Whole blood was diluted 1 : 10 with serum-free RPMI-1640 medium (penicillin 100 U/mL, streptomycin 100 *μ*g/mL) (i.e., 540 *μ*L whole blood diluted in 4.86 mL RPMI-1640 medium), plated in duplicate on 24-well plates at a final volume of 600 *μ*L, and cultured at 37°C in a humidified incubator (5% CO_2_) as described by Wan et al. [28]. Samples were stimulated with the TLR4 agonist LPS (1, 10 ng/mL) and TLR2 agonist Pam3Cys-SK4 [29] (1, 10 ng/mL) and supernatants were harvested after 24 h via centrifuge at 2000 g for 15 min at 4°C. Samples were then stored at −80°C before batch analysis of TNF-*α*, IL-6, and IL-1*β* via ELISA according to the manufacturer's instructions. Biological replicates were analyzed, with the average coefficient of variation (CV) for each cytokine being <5%.

### 2.12. PBMCs Isolation

PBMCs were isolated by gradient density centrifugation of peripheral blood using Ficoll-Paque Plus as described previously by our laboratory [28]. Briefly, 5 mL of blood was layered onto 5 mL of Ficoll-Paque Plus in a sterile 15 mL tube and was centrifuged for 15 min at 800 g and at 20°C. The layer of PBMCs was recovered and washed three times with sterile PBS for 10 min at 250 g at room temperature. Isolated PBMCs were then stored at −80°C until further protein expression analysis by western blotting.

### 2.13. Western Blotting

Proteins from isolated PBMCs were extracted. The protein expression of TLR2 and TLR4 was determined by western blotting following the methods published by our laboratory previously [30]. Signals were visualized using Immobilon western chemiluminescent HRP substrate and bands were quantified by densitometry. Beta actin was used as an internal control.

### 2.14. Statistical Analysis

All data are presented as mean ± standard error of the mean (SEM). Statistical analyses were performed with SPSS version 15.0 for Windows (IL, USA). Data were analyzed for normality and homogeneity before statistical test. Two-way ANOVA was utilized for comparisons between groups. Tukey's Honestly Significant Difference (HSD) was applied for post hoc comparisons. Statistical significance was set at *p* < 0.05.

## 3. Results

### 3.1. Participants' Flow and Participation Rate

**Figure 1:**
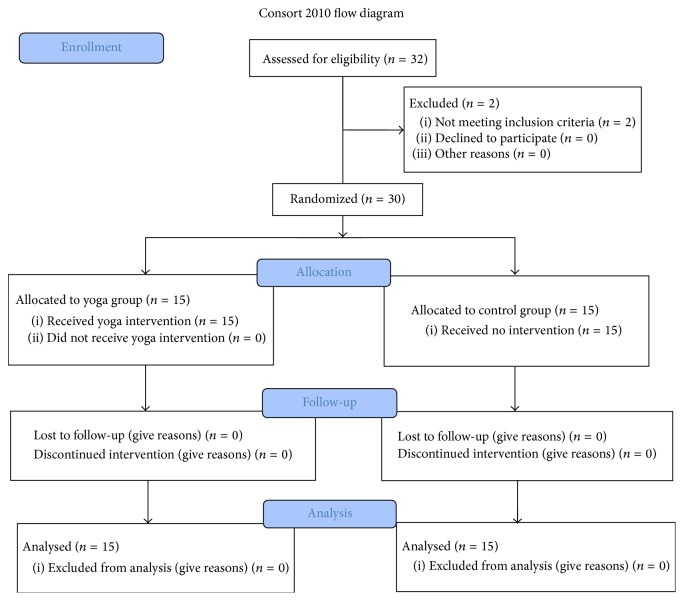
CONSORT flowchart.

The CONSORT flowchart of subject recruitment and intervention was shown in Figure 1. From March 2015 to June 2015, all recruited subjects completed the whole yoga practice with no dropout. There were no harmful effects observed by the yoga practice.

### 3.2. Yoga Practice Decreased Plasma Insulin, Total, and LDL-Cholesterol Level

A total of 8 wks yoga practice resulted in significant reduction in plasma insulin, total cholesterol, and LDL-C levels compared to preyoga practice; meanwhile, HOMA-IR from yoga group is reduced compared to both yoga groups at baseline level and control group after intervention, while there is no difference for glucose, TG, HDL-C, SBP, DBP, body weight, and BMI before and after yoga practice between groups (Table 1).

### 3.3. Yoga Practice Reduced Circulating CD31+/CD42b− EMPs

**Figure 2:**
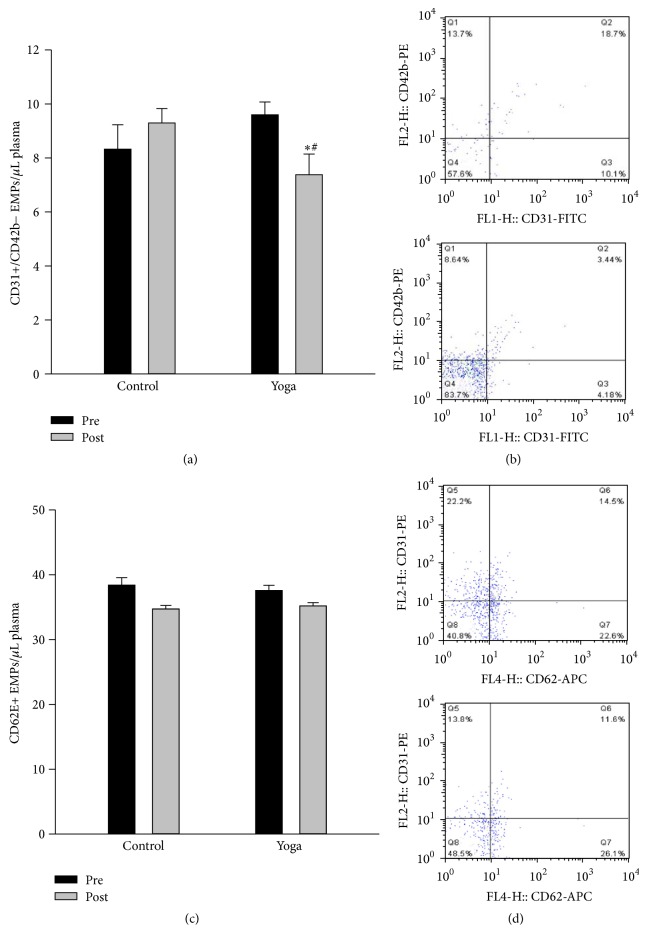
Yoga practice reduced circulating CD31+/CD42b− EMPs levels. Circulating EMPs were measured in platelet-poor plasma by flow cytometry with fluorochrome labeled antibodies specific for PE-CD42b, FITC-CD31, and APC-CD62E. EMPs were identified as CD62E+ and CD31+/CD42b− events with a diameter <1 *μ*M. (a) Fasting CD31+/CD42b− EMPs were reduced postyoga* practice* compared to preyoga training condition. (b) Representative fluorescence-activated cell sorter dot plots of CD31+/CD42b− of a subjects before (top) and after (bottom) yoga* practice*. (c) No difference for CD62E+ EMPs between groups. (d) Representative fluorescence-activated cell sorter dot plots of CD62E+ of a subjects before (top) and after (bottom) yoga* practice*. Data are presented as mean + SEM (*N* = 15). ^*∗*^ *p* < 0.05  versus preyoga training condition within the same group in (a); ^#^ *p* < 0.05 versus control group at baseline level.

As shown in Figure 2, there was a significant reduction in circulating CD31+/CD42b− EMPs after yoga intervention compared to yoga group at baseline level and control group (Figures 2(a) and 2(b)), while yoga practice had no effect on CD62E+ EMPs (Figures 2(c) and 2(d)).

### 3.4. No Effect of Yoga Practice on Circulating Proinflammatory Cytokines

As shown in Table 2, there were no significant effects of yoga practice on levels of plasma proinflammatory cytokines (IL-8, MCP-1, and TNF-*α*) measured in the fasted state.

### 3.5. Yoga Practice Resulted in Decreased Proinflammatory Cytokine Response

**Figure 3:**
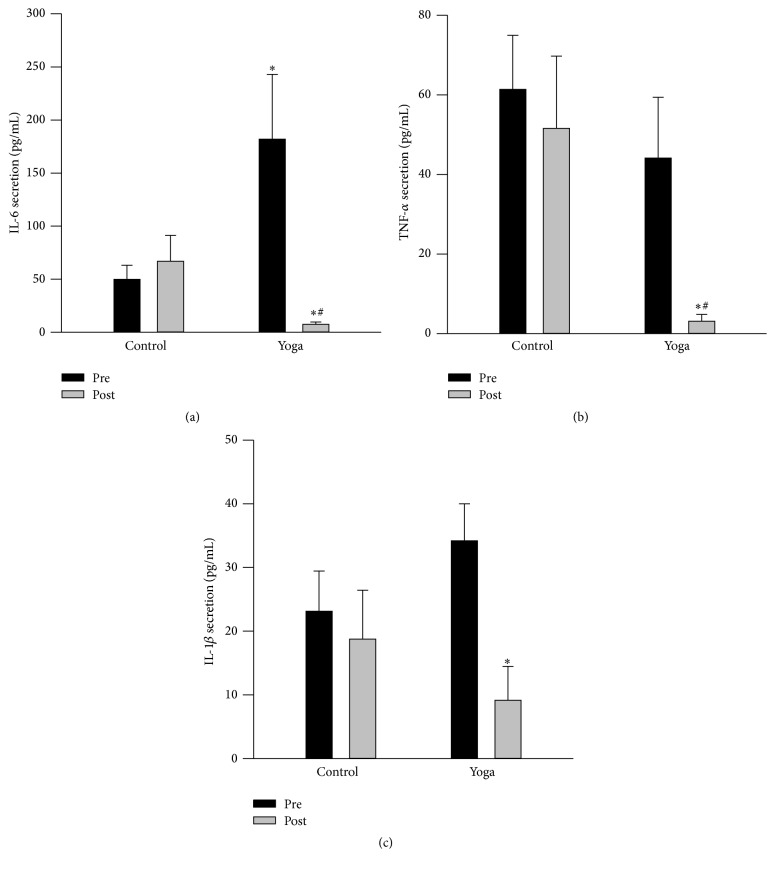
Reduced secretion of IL-6, TNF-*α*, and IL-1*β* from cultured whole blood* ex vivo* after yoga training. Whole blood was collected at baseline and after yoga; then blood was diluted and cultured at 24-well plates under identical culture conditions. Supernatants were centrifuged and collected at 24 hr for the measurement of IL-6, TNF-*α*, and IL-1*β* secretion via ELISA. There is significant reduction of IL-6 (a), TNF-*α* (b), and IL-1*β* (c) secretion after yoga compared to preyoga condition. Data are presented as mean + SEM (*N* = 15). ^*∗*^ *p* < 0.05 versus preyoga training condition within the same treatment; ^#^ *p* < 0.05 versus control group at baseline level.

**Figure 4:**
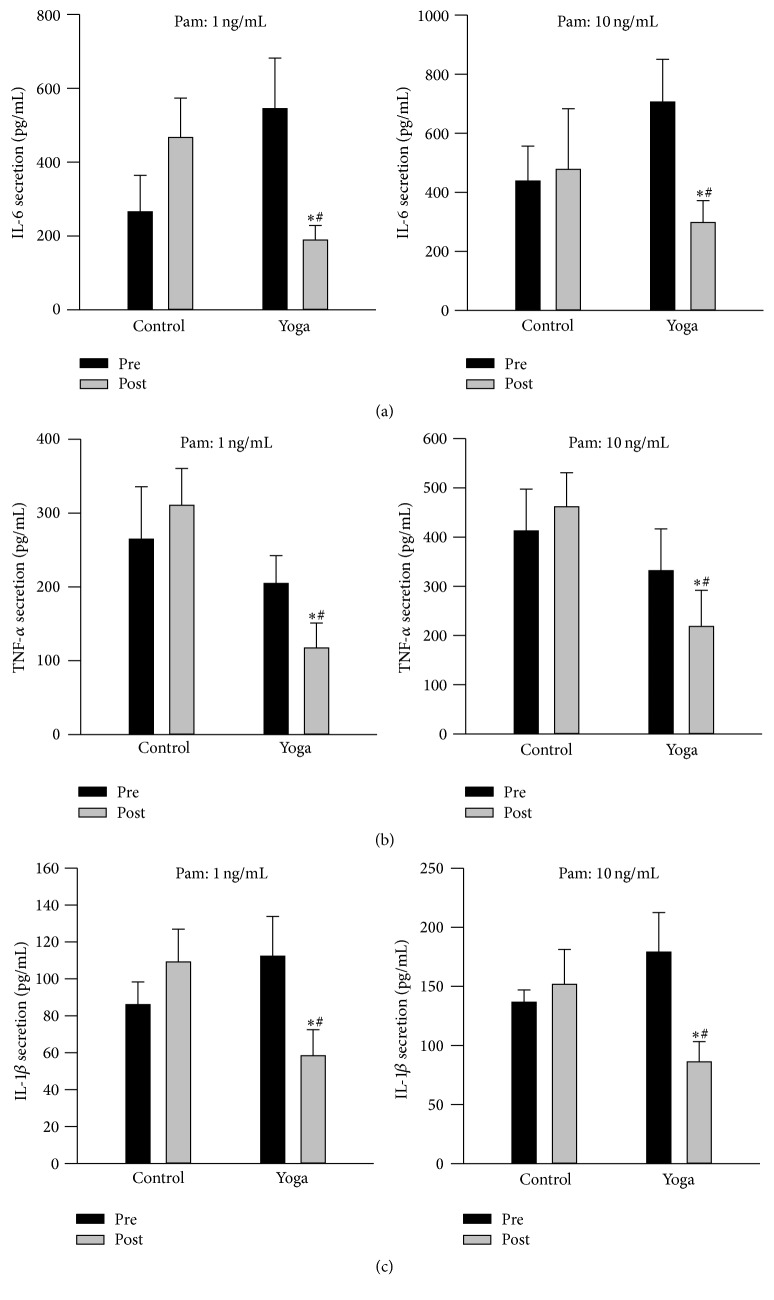
Attenuated Pam3Cys-SK4 (1 and 10 ng/mL) induced IL-6, TNF-*α*, and IL-1*β* secretion from* ex vivo* whole blood cultures after yoga practice. Whole blood was collected at baseline and after yoga; then blood was diluted, cultured at 24-well plates, and stimulated with Pam under identical culture conditions. Supernatants were centrifuged and collected at 24 hr for the measurement of IL-6, TNF-*α*, and IL-1*β* secretion via ELISA. Yoga training led to blunted IL-6 (a), TNF-*α* (b), and IL-1*β* (c) secretion upon Pam stimulation at both 1 and 10 ng/mL. Data are presented as mean + SEM (*N* = 15). ^*∗*^ *p* < 0.05 versus preyoga* practice* condition within the same treatment; ^#^ *p* < 0.05 versus control group at baseline level.

**Figure 5:**
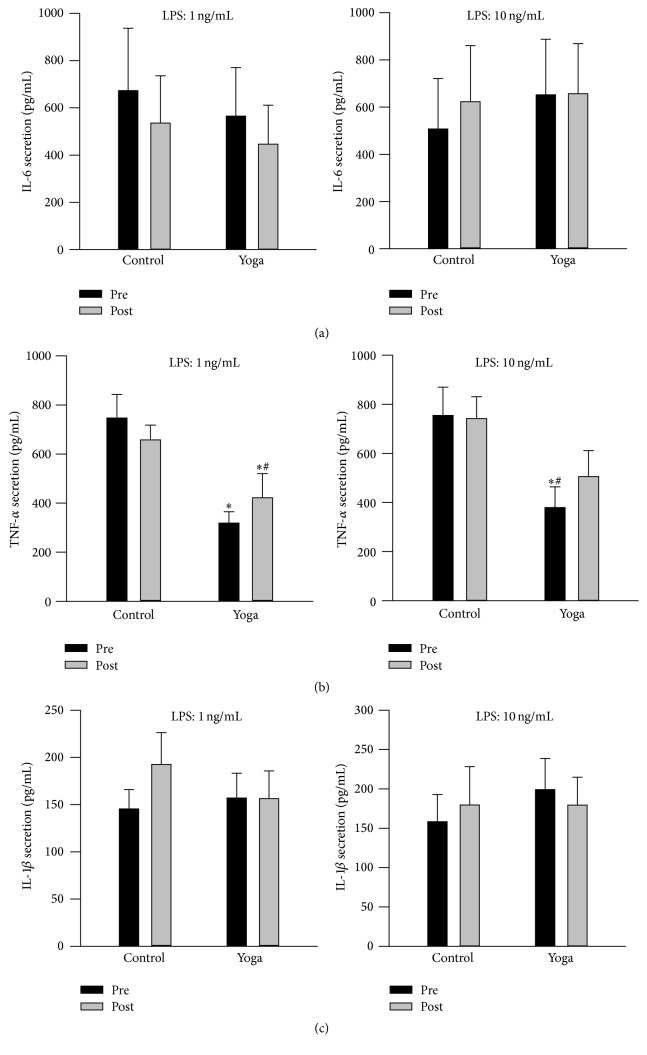
Reduction in TNF-*α* secretion from yoga group compared to control group at baseline and when stimulated with LPS. IL-6 (a), TNF-*α* (b), and IL-1*β* (c) secretion from groups. ^*∗*^ *p* < 0.05 versus control group at baseline level; ^#^ *p* < 0.05 versus control group postyoga practice condition.

At baseline level, yoga group demonstrated elevated IL-6 secretion in supernatant from cultured whole blood at unstimulated condition (Figure 3(a)). Yoga group had reduced secretion of IL-6, TNF-*α*, and IL-1*β* levels after yoga training (Figure 3). Furthermore, when cultured blood was challenged with Pam at both 1 ng/mL and 10 ng/mL, a well-known agonist of TLR-2 receptor [29], yoga practice group also demonstrated damped cytokines secretion including IL-6, TNF-*α*, and IL-1*β* levels compared to preyoga condition and control group (Figure 4). Meanwhile, at baseline level, yoga group has reduced TNF-*α* secretion compared to control group when stimulated with LPS (at both 1 ng/mL and 10 ng/mL); this trend was maintained after yoga training (Figure 5(b)). There is no difference for IL-6 and IL-1*β* secretion when stimulated with LPS (Figures 5(a) and 5(c)).

### 3.6. Yoga Practice Resulted in Decreased TLR2 Protein Expression in PBMCs

**Figure 6:**
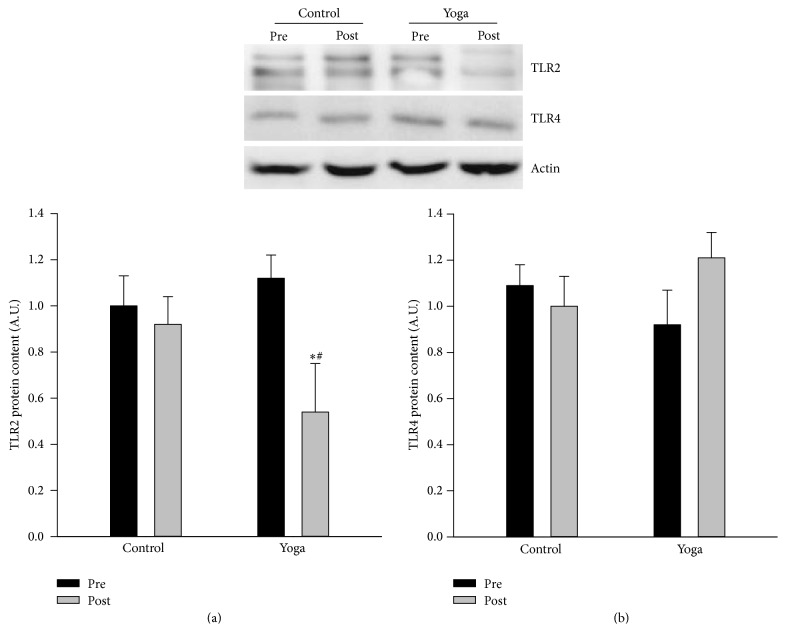
Reduction in TLR2 protein expression from PBMCs after yoga practice. PBMCs were isolated at baseline level and after yoga training, and the protein expression of TLR2 and TLR4 was measured via western blotting. Yoga practice resulted in reduction in TLR2 protein expression with no effect on TLR4. Western blotting images are given at the top of the quantified data. ^*∗*^ *p* < 0.05 versus preyoga training group; ^#^ *p* < 0.05 versus control group at baseline level.

As shown in Figure 6, there is no difference for TLR2 protein expression between groups at baseline level; yoga practice resulted in significant reduction in TLR2 protein expression in PBMCs, while there is no difference for TLR4 protein expression between groups before and after yoga practice.

## 4. Discussion

The main findings of the present study are that (1) 8 wks of Hatha yoga practice in healthy Chinese female subjects reduced plasma insulin, cholesterol levels, and circulating CD31+/CD42b− EMPs and that (2) cultured whole blood from yoga practice group had reduced proinflammatory cytokines secretion at unstimulated condition, as well as when stimulated with a TLR2 agonist, and this might be associated with reduced TLR2 protein expression after yoga training.

The most significant risk factors for MetS include dyslipidemia, hypertension, and physical inactivity [31]. Yoga practice improved lipid profiles in clinical patients with cardiovascular diseases [32, 33] and hypertension [34]. In particular, Bijlani et al. [34] reported that the TG-lowering effects of yoga were more prominent in subjects with hypercholesterolemia [34]. Therefore, when assessing yoga's effects on improving lipid profiles, it is important to consider participants' health conditions. Our present study confirmed that, in healthy, female Chinese subjects, 8 wks of Hatha yoga practice (2 times/wk) could reduce total cholesterol and LDL-C level, indicating that Hatha yoga practice is an effective way for reducing risk factors associated with disordered lipid profiles even in healthy subjects. Randomized trials [35] and meta-analyses [36] have consistently demonstrated a modest but consistent reduction in blood pressure following yoga practice. However, we observed no alterations in SBP and DBP after 8 wks of yoga practice. This might be related to multiple factors. First, different yoga practice type, length, and frequency might affect its effects on blood pressure. Second, the subjects in our present study are healthy; thus it might be hard to observe reductions in blood pressure.

Yoga has been increasingly accepted as a cost-effective therapeutic strategy for T2DM patients [2, 37]. Evidence in regard to how yoga practice affects plasma insulin level remains inconsistent. Hunter et al. [13] reported that Bikram yoga, which is one of the most popular forms of hot yoga, resulted in reduction in plasma insulin and HOMA-IR only in older adults (53 ± 2 yrs). Vizcaino [38] demonstrated that 6 wks of Hatha yoga (3 times/wk) has no effect on fasting insulin level in patients with T2DM. In contrast, Manjunatha et al. [5] reported that yoga practice reduced serum insulin level in healthy subjects, while the majority of them were male. Our present study further confirmed that in healthy female subjects 8 wks of Hatha yoga could significantly reduce plasma insulin level and consequently HOMA-IR.

Elevation of EMPs is rapidly being accepted as an alternate surrogate marker of CVDs and endothelial function [39]. CD62E+ EMPs generally reflect endothelial activation or inflammation whereas CD31+/CD42b− EMPs are released upon endothelial cell apoptosis [7]. Recent evidence has confirmed that moderate-intensity endurance training could reduce circulating EMP levels [11, 40, 41]. In contrast, physical inactivity via reducing daily PA [12] or subjecting subjects to 7 days of dry water immersion [10] is associated with increased concentrations of CD31+/CD42b− EMPs and CD31+/CD41− EMPs, respectively. Our study is the very first to reveal that 8 wks of Hatha yoga could significantly reduce plasma CD31+/CD42b− EMPs in healthy subjects. High concentrations of EMPs are associated with a proinflammatory and antiangiogenic status in the vascular system [42]; thus reduction of CD31+/CD42b− EMPs after yoga suggested that yoga might improve vascular function via affecting EMPs levels. Furthermore, Jenkins et al. [27] reported that an acute reduction in shear stress* via* disturbed blood flow increased local concentrations of CD31+/CD42b− and CD62E+ EMPs in the human forearm. Our result could also suggest that, unlike pathological stress, physiological stress like yoga may decrease EMPs release. This might be one of the mechanisms through which yoga intervention exerts its cardiac and vascular protective effects. However, further studies are required to confirm this hypothesis.

Improved circulating inflammatory markers after yoga practice have been observed in clinical patients with heart failure [15, 16], breast cancer [17, 18], chronic inflammatory diseases, and overweight/obese subjects [19]. In our present study, although yoga practice had no effect on circulating IL-8, TNF-*α*, and MCP-1 levels in healthy subjects,* via* whole blood culture* ex vivo*, reduction in IL-6, TNF-*α*, and IL-1*β* secretion has been observed after yoga training. The whole blood culture method is based on an optimal dilution of the blood cells in medium and no unphysiological cell separation is involved; thus it represents a physiologically much more relevant environment for the cells. Our findings could suggest that yoga practice may reduce the inflammatory status at the whole blood culture level. It is possible that a longer-term yoga practice than the present study design is required to reduce circulating proinflammatory cytokines in healthy subjects. Furthermore, yoga group also demonstrated reduced IL-6, TNF-*α*, and IL-1*β* secretion following TLR2 agonist stimulation but not TLR4; this was also associated with reduced TLR2 protein expression in PBMCs after yoga intervention. Collectively, it is suggested that yoga practice could result in blunted TLR2 response. We are yet to determine whether a yoga-induced blunting of TLR2 response represents a positive change for the health status in the long run. Considering that chronic inflammation is one of the key mechanisms involved in the pathogenesis of MetS [14], in the long term, a decrease in TLR2 response may exert a beneficial effect because it decreases the inflammatory capacity of inflammatory cells, consequently suppressing whole body chronic inflammation. Compared with the reported effects of endurance training on TLR4 expression in men [23], the lack of LPS induced IL-6 and IL-1*β* secretion, as well as no alteration in TLR4 protein expression after yoga practice in our present study, may be related to differences in the type of intervention performed (aerobic, resistance exercise versus yoga), the intensity of the intervention, and/or the population examined. Clearly, more mechanistic studies are required to explore how different types of yoga practice affect TLRs expression and/or function in immune cells not only in healthy subjects but also in subjects with MetS.

## 5. Limitations

Our study has several limitations. First, the population used in our study was small and young healthy female subjects, limiting its generalizability to other populations. Second, the technique for the measurement of EMPs has yet to be standardized, so comparisons across studies may not be appropriate. Third, although we have shown IL-6 and IL-1*β* levels from cultured whole blood, the circulating IL-1*β* and IL-6 levels were below detection limits as measured via Luminex® technology. We acknowledge that it may be difficult to fully compare all of the cytokine markers measured due to differences in measurement technique and the physiological source of the biomarkers.

## 6. Conclusions

A total of 8 wk Hatha yoga practice in healthy Chinese female subjects could improve markers related to MetS, including reduced fasting circulating insulin, cholesterol and LDL-cholesterol levels, and circulating CD31+/CD42b− EMPs, as well as reduced TLR2 response from whole blood culture. As yoga seems to be a relatively safe intervention, it can be considered as an ancillary intervention in the primary MetS prevention for healthy population.

## Figures and Tables

**Table 1:** Comparison of metabolic characteristics between groups before and after yoga intervention.

	Control		Yoga	
	Pre	Post	Pre	Post
Insulin (mIU)	6.17 ± 0.60	5.55 ± 0.75	6.58 ± 0.98	4.06 ± 0.87^*∗*^
Glucose (mM)	4.59 ± 0.07	4.51 ± 0.08	4.59 ± 0.13	4.48 ± 0.1
HOMA-IR	1.26 ± 0.12	1.13 ± 0.17	1.36 ± 0.21	0.75 ± 0.18^*∗*,#^
TG (mM)	0.60 ± 0.06	0.60 ± 0.04	0.66 ± 0.03	0.68 ± 0.09
Cholesterol (mM)	3.90 ± 0.18	3.64 ± 0.15	4.13 ± 0.12	3.75 ± 0.15^*∗*^
LDL-C (mM)	1.93 ± 0.15	1.76 ± 0.13	2.14 ± 0.11	1.81 ± 0.13^*∗*^
HDL-C (mM)	1.69 ± 0.07	1.68 ± 0.05	1.67 ± 0.05	1.58 ± 0.05
SBP (mmHg)	108.0 ± 2.7	105.9 ± 1.5	106.8 ± 2.1	102.5 ± 2.3
DBP (mmHg)	76.15 ± 1.8	72.62 ± 1.8	74.77 ± 2.4	71.83 ± 2.00
Body weight (kg)	54.08 ± 1.65	53.81 ± 1.68	53.35 ± 1.53	52.71 ± 1.57
BMI (kg/m^2^)	20.68 ± 0.46	20.18 ± 0.46	20.55 ± 0.52	20.49 ± 0.52

TG: triacylglycerol; LDL-C: low density lipoprotein-cholesterol; HDL-C: high density lipoprotein-cholesterol; SBP: systolic blood pressure; DBP: diastolic blood pressure; and BMI: body mass index

Data are expressed as mean ± SEM. ^*∗*^Compared with preintervention baseline level; ^#^compared with control group after intervention.

**Table 2:** Plasma cytokines measured in the fasted state before and after yoga intervention.

	Control		Yoga	
	Pre	Post	Pre	Post
IL-8 (pg/mL)	6.17 ± 0.61	5.13 ± 0.52	5.64 ± 0.56	5.03 ± 0.34
MCP-1 (pg/mL)	159.70 ± 20.86	148.95 ± 20.00	145.71 ± 19.95	149.52 ± 14.44
TNF-*α* (pg/mL)	2.15 ± 0.30	2.36 ± 0.38	1.66 ± 0.19	1.96 ± 0.30

Data are expressed as mean ± SEM.
